# Pensions for Old Age

**Published:** 1893-03-11

**Authors:** 


### Pensions for Old Age.

### I.—THREE SCHEMES CONSIDERED.

The question of providing a State pension for old age has been long before the public, but it is doubtful whether any general realisation has been attained of the existing need for such a provision. The statistics of the Poor Law furnish, it is true, conclusive evidence of this need, but there is a tendency to look upon statistics with suspicion. Certainly, they fail to appeal to the hearts and consciences of most of us. Yet it is startling to be reminded that out of every 100 men and women in England who live to the age of 65, 25 at least sink into pauperism, receiving from the parish either indoor or outdoor relief. And these figures do not by any means represent the case in its strongest aspect. Large numbers of the aged are provided for in homes of various descriptions, and (it must be said) degrees of dreariness; a small proportion are aided by private pension funds to keep up their homes, while there remain thousands of old people in our midst, fighting still the battle of life for themselves, without hope of any brighter day, and enduring hardships of pain, hunger, and cold which are little short of heroic in the effort to escape the house. The brave struggle is continued, perhaps, for years; then comes illness or the death of some benefactor, and the precarious hold on the outside world is loosened, and one more blue-clothed figure is added to the listless group which wears out its days in the dreaded refuge. Why dreaded? Excellent beds, warm fires, food above reproach, officials for the most part kindly, and even the variation of an occasional concert or entertainment—such are the characteristics of many, if not most, of the workhouses of to-day. Yet it remains true for ever that "the life is more than meat and the body than raiment." Who can cheerfully face the prospect of spending the last years of life without honour and without affection? The bitterest cares of poverty may be faced with content rather than the loss of all the little services rendered and received, which lend old age its beauty and dignity. And it is the special honour of human nature that this is so. Excluded from all the brightness and warmth of young life the aged paupers wear out their lives in a cold reticence, not wanting in justification, side by side with those who, like themselves, are branded as failures. Those are happiest in whom, as age advances, the animal nature triumphs until a dull content in the mere provision of food and warmth prevails. But is this, indeed, all for which man is fit at the close of his wage-earning days? If the value of life consists in something higher than the power of adding coin to coin, then the system by which we rigidly exclude the aged from their share of duty and privilege is a more serious waste to the country than even the enormous sums which are required to sustain these now useless lives.

Mr. Spender, in a valuable chapter in his book on "Old Age Pensions," has accumulated a vast amount of evidence to show that under present circumstances it is practically not in the power of the working classes to make competent provision for their old age. The existing friendly societies cannot meet the need, since the voluntary contributions of workmen suffice only to ensure sick pay, and a necessary grant to the widow in case of death. Attempts at superannuation allowances are found gravely to imperil their financial position. Mr. Spender further shows that the provision afforded by relatives is unsatisfactory, since the maintenance of parents is a serious drag upon the younger generation, frequently rendering it impossible for a man to save anything for his own old age. It is, moreover, an unnatural and precarious state of things, altogether unusual among the middle and upper classes, and tending ever since the days of King Lear to produce a sentiment akin to antagonism in those upon whom the burden falls.

Since 1865 the Post Office has offered facilities for the buying of annuities. The number of persons who avail themselves of the privilege of State security for their pension has always been small, and is steadily decreasing. The reasons for the failure are probably the high rate charged by the authorities, and the extremely complicated method of effecting the insurance.

More recently three annuity schemes, connected with the names of Canon Blackley, Mr. Chamberlain, and Mr. Charles Booth have been before the public. These must be briefly examined.

Canon Blackley's scheme is based upon the compulsory payment by every man and woman in the kingdom of the sum of £10 before reaching the age of 21. For this payment, each person of the wage-earning class is to be entitled to a sum of 8s. a week during illness, and a pension of 4s. a week at the age of 70. The principal objections to this are: 1st, that the payments are not sufficient to guarantee the benefits offered, on a financially sound basis, since the combination of sick pay with superannuation allowance leaves the amount of the outgoings a matter of absolute uncertainty; 2nd, that the payment, small as it is, could almost certainly not be contributed by the very class of irregular wage-earners who stand most in need of future provision; 3rd, that the scheme involves the levying of a kind of compulsory charity from the non wage-earners, which would be distasteful to the one class, and possibly demoralising in its effects upon the other.

Mr. Chamberlain's scheme offers considerable inducements to the respectable workman earning good wages to make provision for the future. The sum of £5, paid before the age of 25, and subsequent payments of £1 yearly, is to ensure a weekly pension of 5s. at the age of 65, and an allowance for widows and children in case of death before that age. Mr. Chamberlain does not anticipate that the amount paid will be sufficient to cover the proposed liability, but he suggests that the State shall furnish a bonus of £15 to each person ensuring when the first payment of £5 has been made; the compound interest of this £20 at 2½ per cent. is to furnish, with the remainder of the contributions, the sum required to guarantee the pension. The objection to this scheme is simply the fact that it cannot touch the very large class who are entirely incapable, from the conditions of their labour, of saving £5 at any period of their life. Another large class, who could, but certainly would not, make this provision for the future must also be reckoned with. On the whole and briefly it may be said of it that it meets a need, but not the need. It must be hailed if it becomes law as a decided step in the right direction, but it would undoutedly leave the great mass of old age misery at present existing unrelieved.

Mr. Charles Booth proposes that the nation shall, as a free gift, and without any previous contribution, furnish to each person, without distinction of class, a pension of 5s. weekly on attaining the age of 65. Two pertinent questions arise in connexion with this proposal. From what source is the money for the grant (estimated variously at from fourteen to twenty-seven millions yearly) to be obtained? And what effect will be ultimately produced on the nation by a measure which would practically convert all persons over 65 into paupers? It may be safely assumed that these considerations will block the way of this bold scheme for some time to come.

It will be seen from this brief review that none of the existing suggestions can claim any large measure of public approval. In a further article some modifications of these schemes will be discussed with a view to gaining some light on the principal difficulties which stand in the way of a just solution of the problem. That the problem is an urgent one affecting the interests of a very large proportion of the population has been shown. No one who has any intimate knowledge of the lives and position of our aged poor in their own homes will contend that they are best off when shut out of the way. But if such experience be wanting to the reader, let him look round and note the radiance of love which makes so many of the aged in happier ranks of life the centre of joy and consolation to the household, and reflect that here indeed

"One touch of Nature makes the whole world kin."

